# A Comparison of Phylogenetic Network Methods Using Computer Simulation

**DOI:** 10.1371/journal.pone.0001913

**Published:** 2008-04-09

**Authors:** Steven M. Woolley, David Posada, Keith A. Crandall

**Affiliations:** 1 Computational Biology Program, Washington University School of Medicine, St. Louis, Missouri, United States of America; 2 Departamento de Bioquímica, Genética e Inmunología, Facultad de Ciencias, Universidad de Vigo, Vigo, Spain; 3 Department of Biology, Brigham Young University, Provo, Utah, United States of America; University of California, Berkeley, United States of America

## Abstract

**Background:**

We present a series of simulation studies that explore the relative performance of several phylogenetic network approaches (statistical parsimony, split decomposition, union of maximum parsimony trees, neighbor-net, simulated history recombination upper bound, median-joining, reduced median joining and minimum spanning network) compared to standard tree approaches, (neighbor-joining and maximum parsimony) in the presence and absence of recombination.

**Principal Findings:**

In the absence of recombination, all methods recovered the correct topology and branch lengths nearly all of the time when the substitution rate was low, except for minimum spanning networks, which did considerably worse. At a higher substitution rate, maximum parsimony and union of maximum parsimony trees were the most accurate. With recombination, the ability to infer the correct topology was halved for all methods and no method could accurately estimate branch lengths.

**Conclusions:**

Our results highlight the need for more accurate phylogenetic network methods and the importance of detecting and accounting for recombination in phylogenetic studies. Furthermore, we provide useful information for choosing a network algorithm and a framework in which to evaluate improvements to existing methods and novel algorithms developed in the future.

## Introduction

Phylogenies are of central importance in testing comparative hypotheses in a wide variety of fields [1]. Yet, at the population level, there are biological phenomena, such as recombination and hybridization that lead to reticulated relationships. Furthermore, at the population genetic level, lower levels of diversity sometimes lead to a lack of phylogenetic resolution and representing this uncertainty is important. Ignoring these issues may lead to erroneous estimation of evolutionary relationships [2] and/or poor estimates of parameters based on those phylogenies [3]. Therefore, a number of approaches have been developed to represent genealogical relationships as reticulating networks, either by explicitly modeling reticulate events or non-explicitly by representing phylogenetic ambiguity or incompatibility [reviewed in 4], [5].

A wide range of network methods are now available and heavily used by researchers in fields as disparate as phylogeography [6], virology [7], and human quantitative genetics [8]. Nevertheless, the ability of these methods to accurately [sensu 9] estimate the true underlying genealogical relationships has not been thoroughly tested (i.e., assessing consistency, efficiency, and robustness). Indeed, we know of only a few such studies. First, Crandall [10] explored a single method (statistical parsimony) relative to maximum parsimony using an empirically generated data set from a known phylogeny of the bacteriophage T7 [11]. He found that the statistical parsimony approach outperformed maximum parsimony when levels of variation were low. More recently, Cassens et al. [12] provided a more extensive evaluation of three different network algorithms (statistical parsimony, median-joining network, and minimum-spanning network) compared to a newly developed union of maximum parsimony trees approach using simulated data over four known tree topologies. They showed that maximum parsimony performed as well or better than the network approaches under all four tree topologies, that the minimum spanning network algorithm often performed significantly worse with a greatly increased number of errors in the estimation, and that the statistical parsimony and minimum spanning network approaches performed significantly worse when the evolutionary history had many missing intermediates. However, neither study investigated the performance of methods under conditions where reticulating relationships would be an expected evolutionary outcome (e.g., under recombination). It is presumably under such conditions where network approaches have an advantage in terms of estimating genealogical relationships.

Indeed, these notable differences among network approaches, coupled with the report of conflicting inferred histories from empirical data [13], invite a more thorough evaluation of network methods. Previous studies were limited by the number of topologies tested and the number of methods compared, due to difficulty in automating the comparisons (as well as the lack of recombination as indicated above). Therefore, we have embarked on a more extensive study that uses computer simulation to evaluate the performance of seven “non-explicit” network approaches: statistical parsimony [SP], minimum spanning network [MSN], split decomposition [SD], NeighborNet [NN], median network [MED], reduced median joining [RMD], and union of maximum parsimony trees [UMP], as well as one discrete method (shrub-gc) [SHB] which explicitly models recombination and gene-conversion. We also included two standard bifurcating phylogenetic approaches (maximum parsimony [MP] and neighbor-joining [NJ]). We explored the effect of a variety of substitution and recombination rates, sequence lengths, numbers of taxa, and models of substitution on the relative accuracy of these network and standard phylogenetic approaches.

## Materials and Methods

**Figure 1:**
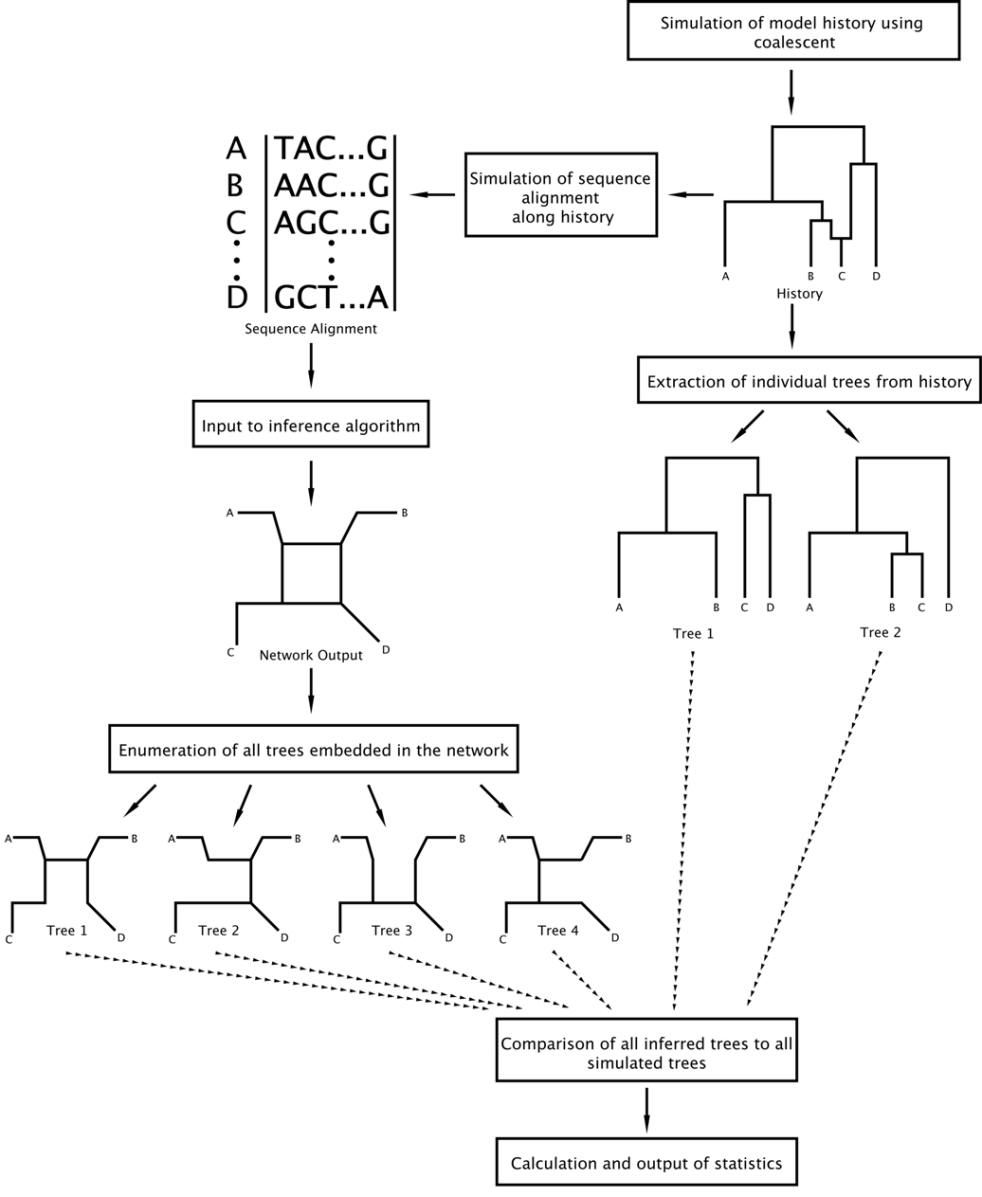
Flowchart outlining our simulation and comparison method.

Our basic approach was to simulate DNA sequences using the neutral coalescent with and without recombination [see 14] and then run the resulting alignments through a variety of algorithms for estimating network relationships among sequences (Figure 1). We then compared the resulting subtrees within these networks to those under the true history and tabulated the frequency of correct subtrees for different approaches. Each of these phases, data simulation, tree processing, performance measures, and estimation approaches explored are detailed below. While simulations can provide general predictions about the behavior of the methods studied, as well as some sense of their robustness (insofar as differing models are explored in the simulations), it is rarely possible to simulate the entire universe of relevant models and the models simulated may represent real data only to a given extent.

### 

#### Data Simulation

DNA sequence alignments were simulated under 18 different sets of conditions (“sets”) selected to represent a range of intraspecific data sets, including some extreme cases. We explored different sequence lengths (500 and 1000 base pairs), numbers of taxa (10, 20 and 50), substitution rates (6.25×10^−6^, 6.25×10^−7^ expected substitutions per site per generation), recombination rates (0, 2.5×10^−5^, 1×10 ^−6^, 4×10^−6^ recombination events per site per generation) under a simple Jukes Cantor nucleotide substitution model [15] with and without a gamma distributed site-rate heterogeneity [16] (Table 1). The substitution rates modeled here are typical for nuclear genes across a diversity of organisms [17]. Recombination rates, on the other hand, have not been widely estimated across loci or organisms. However, in one extensive study examining recombination rates across a diversity of studies involving multi-locus sequence typing from a variety of organisms (and therefore across a range of loci), Pérez-Losada et al. [18] estimated a wide range of recombination rates similar to those modeled here. The effective population size was always 1000. One thousand histories and alignments were simulated under each one of these scenarios to afford reasonable statistical comparisons among the different methods.

**Table 1:** Simulation parameters for the neutral coalescent simulations with and without recombination.

*Parameter Set*	*Sequence Length*	*Number of Taxa*	*Substitution Rate* a	*Substitution Model* b	*Recombination Rate* c	*Mean Number of Haplotypes*	*Mean Number of Unique Histories*
1	500	10	6.25e-7	JC	0	3.390	1
2	500	20	6.25e-7	JC	0	4.160	1
3	500	50	6.25e-7	JC	0	5.260	1
4	500	10	6.25e-6	JC	0	7.540	1
5	500	20	6.25e-6	JC	0	12.220	1
6	500	50	6.25e-6	JC	0	20.550	1
7	1000	10	6.25e-7	JC	0	4.460	1
8	1000	20	6.25e-7	JC	0	5.990	1
9	1000	50	6.25e-7	JC	0	8.200	1
10	1000	10	6.25e-6	JC	0	8.540	1
11	1000	20	6.25e-6	JC	0	15.000	1
12	1000	50	6.25e-6	JC	0	27.830	1
13	1000	20	6.25e-6	JC	0.25e-6	15.054	3.825
14	1000	20	6.25e-6	JC	1.0e-6	15.254	11.85
15	1000	20	6.25e-6	JC	4.0e-6	16.114	40.387
16	1000	20	6.25e-6	JC+Γ	0.25e-6	14.891	3.831
17	1000	20	6.25e-6	JC+Γ	1.0e-6	15.267	11.96
18	1000	20	6.25e-6	JC+Γ	4.0e-6	16.126	39.387

a Substitution rate is expressed in number of substitutions per site per generation.

b In the JC+Γ, α was always set to 0.2.

c Recombination rate is expressed in number of recombination events per site per generation.

#### Tree Processing

In order to make appropriate comparisons between the simulated and inferred trees, branch lengths from the simulated trees were expressed as the number of *realized* changes rather than as the number of *expected* changes. This is because at low substitution rates it is very common that no changes occur along short branches in the simulated trees, and therefore they would be impossible to infer. Moreover, datasets with no realized changes at all were discarded from the analysis. To compare simulated and inferred trees, branches with zero length were collapsed. This was necessary since the tree comparisons described below would consider a zero length branch present in one tree but absent in another as a topological difference.

#### Measures of Performance

Comparing the estimated relationships to the simulated (“true”) underlying relationships is simple when there is no recombination because the simulated evolutionary history is a single tree (sets 1–12, Table 1). However, when recombination is present (sets 13–18, Table 1), the simulated history cannot be represented by a single tree anymore, but by multiple trees that correspond to each of the recombinant fragments. In fact, this set of trees conforms to an ancestral recombination graph [19], [20]. This presents a certain difficulty in that the standard phylogenetic methods will be incorrect, by definition, as they will only return a single tree (or set of bifurcating trees in the case of maximum parsimony).

In order to compare the inferred trees or networks with the simulated trees or networks, we first needed to devise a method for comparing both single trees and sets of trees to single trees and networks. While several metrics have been proposed to compare “idealized” networks (i.e., galled [21], tree-child or tree-sibling [22]), networks estimated from real data (or networks simulated under meaningful models like the coalescent) seldom conform to the restrictions imposed by such representations. For example, in the most general of these, the tree-sibling networks, every hybrid node has at least one sibling that is a tree node; an assumption often violated by empirical data and lacking any evolutionary meaning. Moreover, even under these rather mathematical restrictions, none of these metrics can assure that they only take a value of 0 when two networks are isomorphic, i.e., they are “imperfect” [22]. Another method that has been employed to compare networks with networks or networks with trees is comparison of their list of splits [e.g. 23], which in principle, does not take into account branch lengths, but provides a simple calculation of type I and type II errors. We used related measures, which compare the splits of each tree embedded within a network or tree while accounting for branch lengths associated with each split (see below). We chose to work with tree enumerations or tree lists, regardless of whether the size of these enumerations was just one (for single trees) or more (for networks). For the simulated networks, the coalescent with recombination automatically provides the enumeration of the trees within the simulated network (or ancestral recombination graph). We wanted to measure how often the underlying tree(s) from the simulation (from now on, the “model trees”) were contained somewhere within the inferred tree(s) or network. For this, we used the optimal spanning tree algorithm of Shioura et al. [24], which enumerates all the trees contained in an undirected graph (‘network’) efficiently in terms of computational time and memory. Since the spanning trees from the inferred network may contain inferred internal nodes as tips, they were further processed to remove superfluous internal nodes (unobserved internal nodes with less than three connected branches). Also note that duplicate trees can arise from networks when reticulations can be broken at multiple edges, potentially leaving internal nodes as leaves, which are later pruned such that only input sequences are represented as leaves in the trees. If the number of trees contained within a single network exceeded 5,000,000, that network was excluded from the analysis due to time/resource constraints. Once the set of all trees contained within a network was created, all duplicate trees were removed, leaving only one copy of each tree with a given topology and branch lengths. Furthermore, duplicates were also removed from each set of MP trees (see below). For those methods that give a visual representation of splits (SD, NN, MED and RMD), we used the representation provided, even though that representation may not necessarily be unique. It is not clear whether different representations of the same split system must always embed the same trees.

Once we enumerate the trees contained within the estimated networks, or within the set of trees estimated by the traditional phylogenetic approaches, we need to compare these trees to the model tree(s). We used two related measures for tree comparison, the Robinson-Foulds (RF) score [25] and the branch score (BS) [26]. The RF score is the total number of clades present in one tree but absent in another and does not take into account branch lengths. Therefore, the higher the score, the worse the topological match between the compared trees. The BS, on the other hand, computes the sum of the squares of the differences between each branch's length in each tree. Branches that appear in one tree but not in the other are scored as if compared to a branch of length zero. Two trees are equal when the BS between them is zero (and hence the RF is also zero). Two topologies are the same when the RF is zero. Thus, a tree *t* “exists” in a set of trees *T* if and only if there is a tree *s* in *T* such that the BS between *t* and *s* is zero. Similarly, a topology *t* “exists” in a set of trees *T* if and only if there is a tree *s* in *T* such that the RF distance between *t* and *s* is zero. In addition, we calculated a number of statistics to characterize different aspects of the relative performance of the different approaches. If we let *T* be the enumeration of model trees and *N* be the enumeration of inferred trees, then for each replicate we calculated the:

1. NTR - size of *N* (0, 5×10^6^);
2. FP_TOP_ – fraction of topologies in *N* that do not exist in *T* (0, 1);
3. FN_TOP_ – fraction of topologies in *T* that do not exist in *N* (0, 1);
4. FP – fraction of trees in *N* that do not exist in *T* (0, 1);
5. FN – fraction of trees in *T* that do not exist in *N* (0, 1);
6. Mean branch length difference between matching branches.

where (#,#) indicates the range of each statistic and FP are false positives (type I error) and FN are false negatives (type II error). Additionally, we calculated several other statistics (see Data S1):

- Mean RF score between each tree in *N* and each tree in *T* (0, 1);
- Mean BS distance between each tree in *N* and each tree in *T* (0, ∞);
- Mean RF for false positives and false negatives;
- Mean BS for false positives and false negatives;

For measure 1 above, we calculated the median across all 1000 replicates for each method and simulation scenario. For measures 2–5, we plotted the mean (with Standard Error) across all replicates. Measure 6 is calculated as follows: for each tree *t* in *T* compared with each tree *n* in *N*, for each branch that exists in both *n* (call it *b_n_*) and *t* (call it *b_t_*) (meaning that *b_n_* splits the terminal nodes of *n* into the same two disjoint subsets as *b_t_* in *t*) we compute the mean of *l*(*b_t_*) - *l*(*b*_n_) where *l*(*b*) is the length of branch *b*. However, when interpreting the results of this measure one should remember that this is the average of all branches that were actual matches (meaning that if the method found very few matches, with very similar branch lengths between true and inferred trees, it will appear to do better than if it inferred many of the same branches with larger differences in branch lengths. For measures 6 and *a*–*d* above, we plotted the distribution for the 1000 replicates using box-and-whisker plots displaying the median, first and second quartiles, and outliers (points further than 3/2 times the inter-quartile range of the first and third quartiles). Additionally, we performed a set of paired Mann-Whitney tests to determine whether the results from each method were significantly different. We used an experiment-wise error rate of 0.05 with the Dunn-Sidak multiple test correction.

We also considered several measures designed specifically for networks (both maximum likelihood measures [27] and extensions of a bipartite measure to networks [28]), but each of these requires a rooted phylogenetic network, which only one of the methods we tested (SHB) provides, therefore precluding its use here. Our choice of metric does have some deficiencies (RF tends to reward lack of resolution, since a star tree will receive no penalty for false positives, only penalties for false negatives, see [29]). Unresolved trees are penalized by their false negatives (and thus will have lower accuracy) but highly reticulated networks (i.e., those that imbed numerous trees) will have increased false negatives (due to inferring many incorrect branches). Furthermore, it is perhaps not ideal to give all possible trees imbedded within a network equal weight when comparing two phylogenetic networks (since some trees will be much more likely than others); however, we feel using all imbedded trees captures the essence of both accuracy (whether the simulated history was represented) and precision (how many inferred trees do we have to look at to find the true history), even though the exact number of trees and averages across all imbedded trees should be interpreted carefully with this in mind. Also, due to the poor performance of all methods on the medium and high recombination sets, particularly in inferring correct branch lengths, we included measure 6 above to help distinguish how well the individual branch length estimates compared to the true branch lengths.

Finally, we were also interested in the broad scale effect of the type of data on the performance of each method. We measured the relationship between characteristics of the simulated data and the inferred trees using the Spearman correlation coefficient (ρ). We considered the relationship between the number of inferred trees and the number of unique simulated haplotypes used to infer those trees. In the sets with recombination, we also measured the relationship of the number of simulated trees in *T* with the number of model topologies found. In addition, we measured the relationship between the number of inferred trees and the number of trees simulated in *T* in the recombination sets.

#### Network Methods Evaluated

We evaluated ten different approaches commonly used to infer evolutionary relationships at the intraspecific level, including two traditional bifurcating tree-building approaches and eight network building approaches. The bifurcating tree approaches employed in this study were maximum parsimony (MP) [30] and neighbor-joining (NJ) [31] as implemented in PAUP* v4.b10 [32]. MP was run with “maxtrees” set to 5,000,000 and 1000 random sequence additions and NJ trees were built using uncorrected sequence distances. The implicit network building approaches tested were the union of maximum parsimony trees (UMP) [12] as implemented in the software CombineTrees, statistical parsimony (SP) [33] as implemented in the software TCS v1.17 [34], split decomposition (SD) as implemented in SplitsTree (also known as Jsplits) 4 beta 4 [35], Neighbor-Net (NN) and unreduced median networks (MED) as implemented in SplitsTree4 version 4.7 [36], reduced median-joining (RMD) [37] as implemented in Network version 4.2.0.1 [38] and minimum-spanning network (MSN) [39] as implemented in the software Arlequin v2.001 [40].

The explicit network building method tested seeks to calculate the upper bound on the minimum number of recombination events (and gene conversions) while simultaneously computing the most parsimonious tree, as implemented in the shrub-gc software (SHB) [41]. The CombineTrees software takes as input all inferred MP trees and combines them into a single reticulated network merging branches, tip haplotypes, and interior haplotypes that are identical among all trees. CombineTrees was run as in [12], by randomizing the order of the input trees 10 times and picking the smallest network (*i.e.*, with the least number of branches). (Note this does not necessarily mean the smallest number of loops.) On some datasets, CombineTrees was unable to find a network for some orderings of the input trees. In these instances, we still used the smallest network, although in such instances there were less than 10 from which to choose. SD networks were built using default settings. NN was run with two configurations: first, using all defaults and then using a weight threshold set to the inverse of the input sequence length. In the latter case, splits with low support were not included, resulting in a more refined network, and branch lengths greater than or equal to one. We only included results from the former case, since they were much more accurate, even when accounting for the increase in FN. MED was run with default settings. For SP, the maximum connection limit was ignored, forcing all sequences to be connected in a single network. RMD and MSN were run with default settings. Shrub-gc was also run with default settings, but using as input the set of sites where only one or two nucleotide states was observed, since it requires biallelic site data. The resulting ancestral recombination graphs were converted to a list of trees in two manners. For the zero recombination sets, recombination edges were treated as branches defined by the sites derived from that edge's parent that differed from the other parent. For the simulations with recombination, the recombination edges were treated as defining alternative trees such that any tree could only contain one of the two edges associated with each inferred recombination event, resulting in 2^n^ trees for a network with *n* recombination events (note, some of these trees may not be unique and only one copy of each tree was used in further analyses). For the SD, NN, and NJ methods, branch lengths were multiplied by the number of sites and rounded to the nearest integer.

## Results

### No Recombination

#### Topological Type I Error

**Figure 2:**
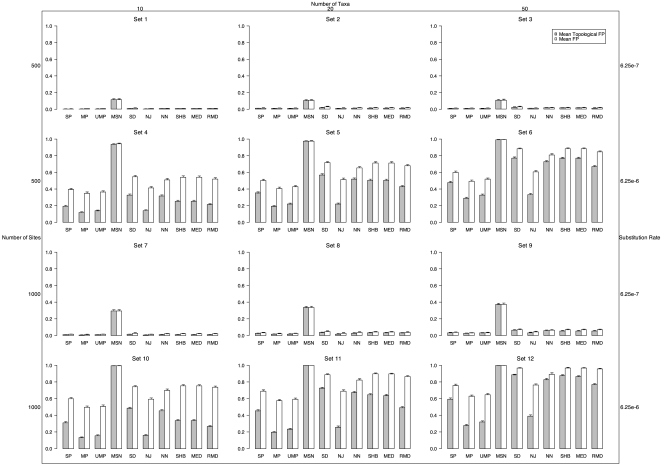
Mean fraction of false positive topologies (FP_TOP_) inferred (those topologies inferred but which did not match the simulated topology) and false positive trees (FP) without recombination. The left margin shows the number of nucleotides in each simulated sequences. The top margin shows the number of sequences simulated. The right margin shows the substitution rate of the sequences.

Topological false positive rate was measured as described above, and the mean over the 1000 replicates per dataset were plotted (Figure 2). All of the methods (except MSN which did noticeably worse) had roughly the same mean topological FP (less than 0.05) with low substitution rates. When the substitution rate was higher, MP had lowest mean topological FP (0.12–0.29), followed by UMP and NJ (0.14–0.39). MSN performed worst, with a mean topological FP always above 0.93. With low substitution rates, the methods performed slightly worse with an increasing number of unique sequences (or haplotypes). This decrease was much greater with higher substitution rates.

#### Tree Type I Error

The mean tree false positive rate (over the 1000 replicates) is shown for each method on each set of data (Figure 2). With a low substitution rate, the mean branch score accuracy of all methods was roughly the same (except for MSN, which once again was significantly higher). All methods but MSN had a false positive rate of less than ∼0.07 with a low substitution rate. The FP rates with higher substitution rates however were much worse. The lowest error rates in this case were achieved by MP (0.35–0.63). UMP had the next lowest mean FP (0.36–0.65), with SP and NJ following (0.40–0.76). The FP rate of MSN was again the worst, always above 0.94. Increasing the number of sequences substantially increased the mean false positives in all methods.

#### Number of trees inferred

The previous two measures give us a sense of how many incorrect trees are inferred by a given method. We also measured the total number of unique trees (NTR) contained within each inferred network and the median of these totals for each method and simulation set (Table 2). At first glance, we can see that some methods generated many more trees than others. This means that while an inference may in fact include the true tree, it may do so by inferring thousands of potential trees. When the substitution rate was low, all methods had a median NTR of 1, while MP inferred fewer trees on average when the substitution rate was higher (except NJ which always infers a single tree). SP and UMP had lower NTR on average on all simulations. When the substitution rate was high, SD, NN and MSN had median NTR above 100 in most simulations, but the remaining methods typically had NTR below 10, except SHB and MED in sets 11 and 12 where they too had significantly higher median NTR. We also found that at the higher substitution rate, several methods (SD, MSN, NN, SHB, MED, RMD) inferred highly reticulated networks (containing more than 5,000,000 trees) on one or more of the simulated histories (Table 2). The number of networks containing this large NTR ranges from a few to over 672 for NN in set 12 (Table 1).

**Table 2:** The median number of trees (NTR) inferred by each method for each set of simulated sequences (number of simulations out of 1000 that were used).

	SP	MP	UMP	MSN	SD	NJ	NN	SHB	MED	RMD
1	1	1	1	1	1	1	1	1	1	1
	(1000)	(1000)	(1000)	(1000)	(1000)	(1000)	(1000)	**(999)**	**(999)**	(1000)
2	1	1	1	1	1	1	1	1	1	1
	(1000)	(1000)	(1000)	(1000)	(1000)	(1000)	(1000)	(1000)	(1000)	(1000)
3	1	1	1	1	1	1	1	1	1	1
	(1000)	(1000)	(1000)	(1000)	(1000)	(1000)	(1000)	(1000)	(1000)	(1000)
4	1	1	1	1	1	1	1	1	1	1
	(1000)	(1000)	(1000)	(1000)	(1000)	(1000)	**(967)**	(1000)	(1000)	(1000)
5	1	1	1	3	4	1	4	1	1	1
	(1000)	(1000)	(1000)	(1000)	**(998)**	(1000)	**(871)**	(1000)	**(996)**	(1000)
6	1	1	1	9	16	1	129	1	1	1
	(1000)	(1000)	(1000)	(1000)	**(989)**	(1000)	**(666)**	(1000)	**(985)**	(1000)
7	1	1	1	1	1	1	1	1	1	1
	(1000)	(1000)	(1000)	(1000)	(1000)	(1000)	(1000)	(1000)	(1000)	(1000)
8	1	1	1	1	1	1	1	1	1	1
	(1000)	(1000)	(1000)	(1000)	(1000)	(1000)	(1000)	(1000)	(1000)	(1000)
9	1	1	1	1	1	1	1	1	1	1
	(1000)	(1000)	(1000)	(1000)	(1000)	(1000)	(1000)	(1000)	(1000)	(1000)
10	1	1	1	3	4	1	4	1	1	1
	(1000)	(1000)	(1000)	(1000)	(1000)	(1000)	**(895)**	(1000)	(1000)	(1000)
11	1	1	1	8	16	1	169	4	4	1
	(1000)	(1000)	(1000)	(1000)	**(985)**	(1000)	**(638)**	**(999)**	**(975)**	(1000)
12	1	1	1	48	244	1	*	14	41	1
	(1000)	(1000)	(1000)	**(988)**	**(917)**	(1000)	**(327)**	**(997)**	**(889)**	**(998)**
13	1	1	1	8	52	1	30861	2	36	4
	(1000)	(1000)	**(997)**	(1000)	**(938)**	(1000)	**(509)**	(1000)	**(842)**	**(993)**
14	1	2	4	4	2183	1	*	9	*	16
	(1000)	(1000)	**(975)**	(1000)	**(775)**	(1000)	**(182)**	**(999)**	**(391)**	**(956)**
15	1	3	72	4	1308	1	*	227	*	1456
	(1000)	(1000)	**(825)**	(1000)	**(821)**	(1000)	**(8)**	**(983)**	**(18)**	**(751)**
16	3	2	4	9	208	1	*	6	3562.5	4
	(1000)	(1000)	**(984)**	(1000)	**(901)**	(1000)	**(76)**	(1000)	**(623)**	**(981)**
17	3	2	9	8	1369.5	1	*	16	*	16
	(1000)	(1000)	**(933)**	**(999)**	**(816)**	(1000)	**(24)**	(1000)	**(286)**	**(941)**
18	4	4	201	8	624	1	*	206	*	1008.5
	(1000)	(1000)	**(781)**	**(999)**	**(843)**	(1000)	**(0)**	**(969)**	**(20)**	**(770)**

Those in **bold** are combinations in which one or more data set contained more than 5,000,000 trees.

* Less than 50% of inferred networks contained less than 5,000,000 so the median NTR cannot be determined.

All methods (except NJ, which infers a single tree in all cases) showed a highly significant positive correlation between the NTR and the number of unique sequences. This was especially true for SD and MSN, with Spearman's ρ = 0.664 and 0.637 respectively. NN, SHB, and MED were slightly less correlated, with ρ = 0.59, 0.527 and 0.509 respectively. For SP, MP, RMD and UMP, the correlation was lower with ρ = 0.356 for SP and ρ = 0.316 for MP, RMD and UMP.

#### Topological Type II Error

**Figure 3:**
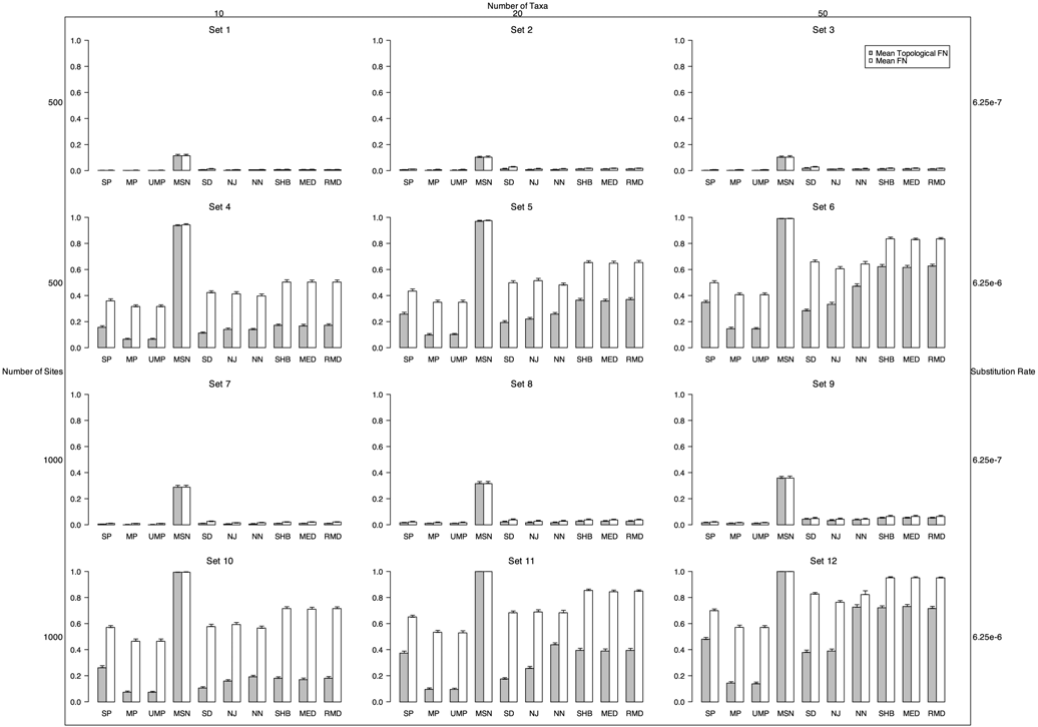
Mean fraction of false negative topologies (FN_TOP_) inferred (fraction of times that the simulated topology was not correctly inferred) and false positive trees (FN) without recombination. The left margin shows the number of nucleotides in each simulated sequences. The top margin shows the number of sequences simulated. The right margin shows the substitution rate of the sequences. Note, the fraction of true positives is 1 - FN.

We also computed the mean topological FN rate and the mean tree FN rate (Figure 3). (Note, the fraction of true positives is simply 1-FN). The mean topological FN with a low substitution rate was not qualitatively different from the topological FP (Figure 2). However, a higher substitution rate resulted in FN patterns different from the FP rates. The first conspicuous but expected result was that all methods (except NJ) had lower FN when compared to FP since there was a single model tree, but all methods (except NJ) could potentially infer more than a single tree, which may not match the model topology (increasing the FP rate). This effect was most dramatic on the SD method, whose mean FP was sometimes double its FN rate. NN also had much larger FP than FN rates with a higher substitution rate.

#### Tree Type II Error

We also calculated the mean tree FN (Figure 3). When the substitution rate was low, the results were again very similar to the tree FP. The most pronounced difference was again the lower mean FN of SD and NN, compared to FP. Smaller increases in FN, relative to FP occurred in all other methods (except NJ, in which FP and FN are by definition equal with only one simulated tree). The tree FN of UMP and MP were the lowest (0.31–0.57), followed by SP (0.36–0.70).

### Recombination

#### Topological Type I Error

**Figure 4:**
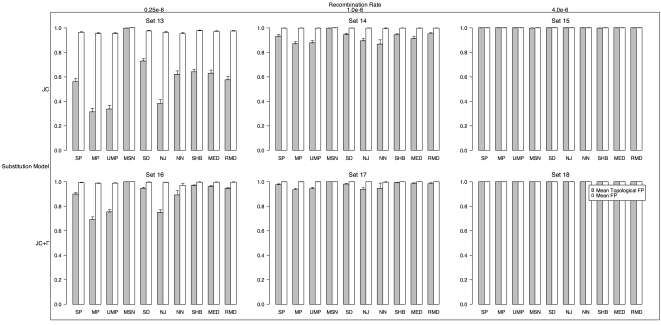
Mean fraction of false positive topologies (FP_TOP_) inferred (those topologies inferred but which did not match the simulated topology) and false positive trees (FP) with recombination. The top row was simulated with a constant substitution rate among sites, while the bottom row was simulated with gamma distributed site-rate heterogeneity. The top margin shows the recombination rate.

With the simulated history potentially containing multiple distinct trees for different sites, we now can potentially recover more than one model tree or topology. In order to evaluate how well the simulated topologies were inferred, we again calculated the mean topological FP rate (Figure 4). Thus, if all inferred topologies were found in the list of true simulated trees for a given method in all 1000 simulations for a particular set of parameters, this value would be 0.0. In the simulations with no rate heterogeneity, MP and UMP exhibited the lowest mean topological FP at 0.31 and 0.34, respectively, for low recombination, but MP, UMP and NN had the lowest topological FP (0.87) with the medium recombination rate. With site rate heterogeneity, the relative topological FP among methods was similar to the constant rate simulations, but all methods had much higher mean topological FP. MP had the lowest FP with both low and medium recombination (0.69 and 0.94 respectively). Notably, all of the methods had mean topological FP rates of 1.0 at high recombination.

#### Tree Type I Error

We also computed the mean tree FP rate in the presence of recombination (Figure 4). It is noteworthy that none of the methods achieved a mean tree FP less than 0.95. In fact, for medium and high recombination rates, on average all methods had tree FP of 1.0. With low recombination, NN had the lowest mean tree FP (0.95 and 0.97 with homogenous and heterogeneous rate variation, respectively).

#### Number of trees inferred

In order to compare the number of trees inferred by each method, Table 2 shows the median NTR for each method on each recombination simulation (sets 13–18, Table 1). As expected, NJ was the lowest, since it always infers one and only one tree. MP and SP on average inferred the second and third lowest NTR respectively, except in set 16 (Table 1), when SHB inferred fewer trees than SP on average. The median NTR for MP and SP was always less than five. The difference between the mean number of trees inferred with MP and SP was not significant in sets 14, 15 or 18 (Table 1). Some of the median values for NTR for NN and MED could not be accurately calculated due to the inability to enumerate all trees within their output (see Table 2).

All methods (except NJ which infers a single tree in all cases, and NN probably due to our inability to enumerate all trees in many of the simulations with recombination) showed a highly significant positive correlation of the number of trees inferred with the number of unique sequences simulated when all recombination sets were analyzed together. The smallest spearman correlation was with NN (ρ = 0.042) followed by SD, with ρ = 0.051 and SHB had the greatest correlation with ρ = 0.351. RMD, MP, MSN, UMP, SP and MED had ρ = 0.218, 0.195, 0.176, 0.155, 0.147, 0.117, respectively. The number of trees inferred by a method when the sequences have undergone recombination should ideally be positively correlated with the number of simulated trees. The association between the number of trees inferred and the number of trees simulated with recombination were statistically significant for all methods tested. SHB had the largest correlation with ρ = 0.829. RMD, MED, UMP, MP, NN, SD and SP had ρ = 0.612, 0.368, 0.304, 0.299, 0.296, 0.167 and 0.123, respectively. Surprisingly, MSN had a ρ = −0.076 (meaning that as the number of trees simulated increases, the number of trees inferred by MSN tends to decrease).

#### Topological Type II Error

**Figure 5:**
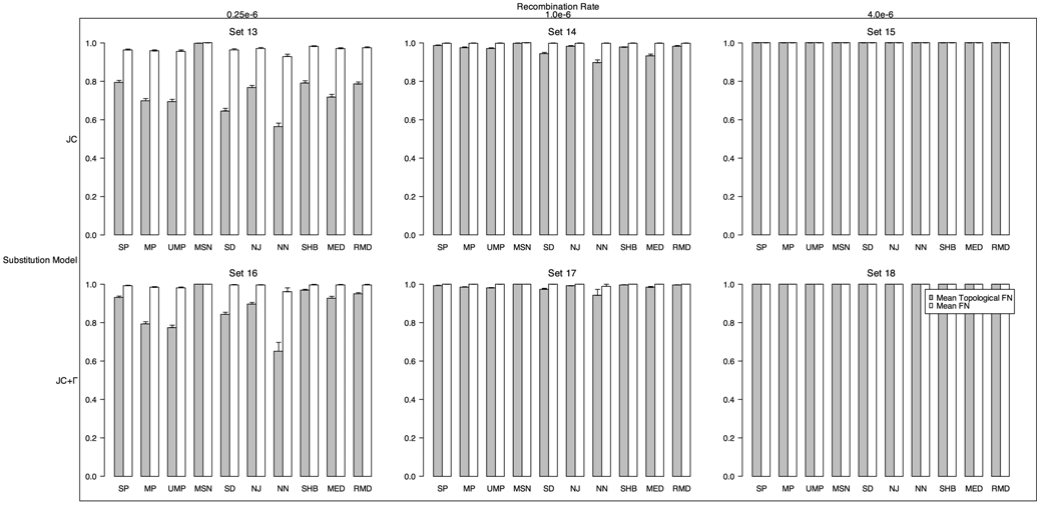
Mean fraction of false negative topologies (FN_TOP_) inferred (fraction of times that the simulated topology was not correctly inferred) and false positive trees (FN) with recombination. The top row was simulated with a constant substitution rate among sites, while the bottom row was simulated with gamma distributed site-rate heterogeneity. The top margin shows the recombination rate. Note, the fraction of true positives is 1 - FN.

In order to assess the fraction of false negative inferences (FN) of each method in finding the simulated topology in the presence of recombination, we calculated the mean topological FN for each method on each simulation set (Figure 5). (Note, the fraction of true positives is simply 1-FN). NN had the lowest mean topological FN in all the low and medium recombination simulations. In the constant site substitution rate simulations, NN had mean topological FN of 0.56 and 0.90 for low and medium recombination respectively and 0.65 and 0.94 with heterogeneous substitution rates among sites. For the highest recombination rate, all methods had mean topological FN of 1.0.

#### Tree Type II Error

Similarly, the fraction of false negative inferences of the simulated trees was calculated. (Again, the fraction of true positives is simply 1-FN). The mean tree FN across each set of 1000 simulations is shown in Figure 5. Mean tree FN was very near 1.0 for all methods tested (over 0.93), and like the topological FN, NN was the lowest with low and medium recombination.

#### Comparison of branch lengths

**Figure 6:**
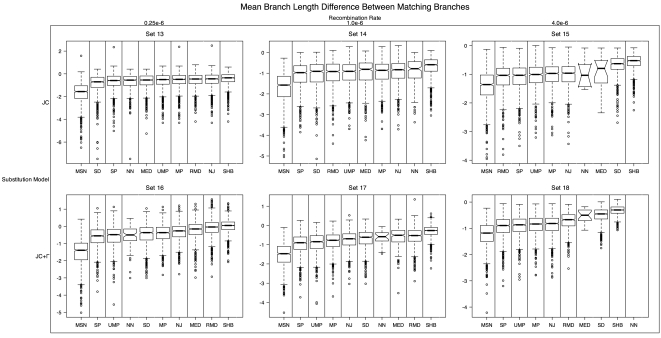
Mean branch length difference between matching branches with recombination. The top row was simulated with a constant substitution rate among sites, while the bottom row was simulated with gamma distributed site-rate heterogeneity. The top margin shows the recombination rate. Vertical lines separate those methods that were significantly different in a paired Mann-Whitney test (see Measures of Performance).

Since the error rates for inferring true trees (both FN and FP) with recombination were so high (see mean tree FN and FP in Figure 4 and Figure 5), we present another view of the accuracy of estimating the branch lengths with recombination. For each branch in an inferred tree that was found to be a match (e.g., induced the same split) in a simulated tree, we calculated the difference between the true branch length and the inferred branch length. We then averaged this value over all matching branches between compared trees and over all 1000 simulations for each set (Figure 6). Thus, if the method consistently overestimates branch lengths, we will have a negative mean, and if it underestimates branch lengths, we have a positive mean. All methods overestimated branch lengths with recombination (Figure 6) and without (see Data S1). The branch lengths estimated by SHB were the closest to the true matching branch's length on average, and had the smallest variance. However, one should be careful interpreting this result, since we can only compare two matching branch's lengths if they actually exist in both model and inference. Thus, a method that had very few matching branches would not necessarily perform poorly in this respect (i.e., if it only inferred one correct branch, with the correct branch length, it would have zero variance and zero mean, even though it would likely have large FP and FN).

## Discussion

### No Recombination

The common use of phylogenetic inference in population studies warrants a thorough analysis of the strengths and weaknesses of network methods. This study was designed to assess the relative performance of ten commonly used network methods on data simulated in a variety of biologically meaningful scenarios. Our analyses have shown that not all methods fare equally well in many circumstances. One important but expected finding is that increasing substitution rate resulted in a significant increase in error (both topologically and in terms of inferring the correct branch lengths) in all methods. Increasing the number of sites also resulted in an increase in mean topological error rates for all methods except MP and UMP when the number of sequences was 20 or 50. When taking into account branch lengths, all methods had increased error as the number of sites increased. We speculate that this decrease in accuracy with an increasing number of sites is a result of the increasing number of unique haplotypes that result from longer sequences. Since we found that an increase in the number of unique haplotypes correlated with an increase in the number of inferred trees, which increases the type II error, we also speculate that with a larger number of sequences to connect there is more uncertainty as to how they are related (and more internal nodes), and thus the error rates are higher. Increasing the number of sequences also resulted in an increase in error for all methods. Overall, MP had at least as low, if not lower error rates than the other methods tested under all circumstances. With low substitution rates, however, the difference in accuracy of MP over UMP, NJ, SP, and SD in general faded away. At higher substitution rates, MP was always significantly less erroneous than all other methods.

One major advantage, however, of the network approaches, is the ability to display ambiguity in the inference in a single graphical representation. MP does not provide such a view, beyond the total number of equally parsimonious trees. However, the method of UMP was designed specifically to facilitate visualization of the set of MP trees in a single graphical representation. The UMP method, by definition, will always result in the same or lower FN as MP with one caveat: increasing the number of trees imbedded in the network may increase the FP rate. This minor limitation is apparent with higher substitution rates when the FP rate is increased in UMP as compared to MP. The accuracy of SD, NN and MSN suffered, although the overall accuracy of the other methods (except NJ) also decreased somewhat due to ambiguity (higher FP rates). It is apparent from the results that MP and/or UMP should be preferred when lower error rates (e.g., higher reconstruction accuracy) is the goal, particularly with relatively divergent sequences. The relative level of topological error as compared to overall tree error was slightly different between all methods, but again, MP and UMP generally had lower error than the rest.

### Recombination

As only one of the inference methods tested (SHB) explicitly accounts for recombination, it is not surprising that the results on the sets simulated with recombination were quite poor. However, even SHB performed poorly in the presence of recombination. Furthermore, as the recombination rate increased, error rates increased to 100% in all methods. When the branch length accuracy was considered (tree FP and FN), no method had mean error below 0.94 (see Figure 4 and Figure 5). This was attributable in part to the difficulty in estimating branch lengths for the entire length of a set of sequences when only certain sites within those sequences actually share the same history, as is the case with recombination. When we only measured each method's topological error, it was much lower, though still not as low as in the simulations without recombination. NN, MP, UMP and NJ were the least erroneous in inferring topology with recombination (see Figure 4 and Figure 5). It was much less clear which method did best when estimated branch lengths in the presence of recombination. In order to better judge the branch length estimates for the methods tested, we calculated the mean difference between branches that matched between the inferred and simulated trees (Figure 6 with recombination and Data S1 for no recombination). This gives us some sense of whether a method consistently over or under estimated the branch lengths. SHB had the smallest mean difference in branch lengths and the smallest variance, which may imply that it does quite well in estimating certain branches accurately, but still can not globally infer all branches in the simulated histories.

Another important consideration in the recombination inferences is the proportion of sites that support a given tree. One might value accuracy in inferring the tree or trees that underlie a large number of sites over one that is only representing a few sites. Trees could be weighted based on this to achieve a more useful measure of accuracy, penalizing a method more for not finding those trees that are supported by a majority of the sites, for example. This should be an area of additional focus in future comparisons and benchmarking of new methods. However, our results indicate that estimates of branch lengths from data with recombination should not be relied upon, at least at the level of the full tree. In addition, rate variation increased the error of all methods significantly. While these results do not look promising for inferring histories from sequences that have undergone recombination in their history, they certainly highlight the importance of detecting recombination within a sample of sequences before confidence is placed on any histories inferred using these methods. Alternatively, methods that explicitly account for recombination during inference could be used, although SHB as tested here showed no general advantage over the other methods (although the strong correlation between the number of trees inferred by SHB with the number of unique simulated histories in the ARG and lower individual branch length inference error, do give some hope for better characterizing its sources of error).

### Conclusion

The method that was consistently the least erroneous in our simulations was MP and the related UMP method. While, nearly all methods exhibited similar performance on sequences with low substitution rates, MP and UMP outperformed the other methods in terms of both lower topological and overall tree error in nearly every case. The development of the UMP method to combine maximum parsimony trees into a single network appears to be quite appropriate. Particularly, if the UMP method can be refined in such a way as to 1) not depend on the order of the input trees, 2) not choke on particular sets of trees ordered in a particular manner, 3) reduce the ambiguity to only that ambiguity existing in the input trees and 4) express the confidence of particular branches within the combined network, it looks very promising for the accurate estimation and visualization of intraspecific phylogenies. While there were some instances where UMP inferred highly reticulated networks on the simulations with recombination, it was not as common as with NN or SD (see Table 2).

As for the other methods tested, the biggest drawback for SD and NN was their highly reticulated representations and their less accurate estimation of branch lengths. However, NN did have slightly lower FN in the recombination sets, indicating that it may still have some potential to capture correct relationships. Since both SD and NN aim to represent the compatible splits in the sequence data, resolution is not necessarily their primary goal, but our results indicate that quite frequently the model tree is not included within their representations, a finding that needs closer inspection.

RMD, MED, and SHB performed fine with low substitution rates, but were significantly less accurate than the best methods when the substitution rate was higher. SHB, in spite of being designed to deal with recombined sequences, performed poorly, even in sets with recombination, although the number of trees it inferred was highly correlated with the number of unique trees simulated in the ancestral recombination graph and its average branch length estimation with recombination was promising. SHB's increased error rates might be due in part to its requirement for binary state alleles as input, reducing the amount of information available for reconstruction.

NJ performed marginally well, although its obvious drawback is its inability to represent ambiguity, either by reticulations, or by inferring multiple trees. This could possibly be addressed by building NJ trees from various partitions of the alignment, and combining the results in a manner similar to UMP, or by including ties or suboptimal NJ trees [e.g. 42], although NN also uses an agglomerative approach similar to NJ, but appears to do worse than NJ in most of our simulations.

SP's performance was not as good as the tree approaches (MP, UMP and NJ) under higher substitution rates, but in most of our simulations, it had lower error than the other network methods (except for topological FN). This gives us hope for improvement, particularly with these benchmarks on which to assess its deficiencies. One possible reason for the method's lower accuracy could be the effect of ignoring the parsimony limit and forcing the software to connect all sequences. This act violates the theoretical advantage of SP over MP, but was necessary in order to compare the performance of all methods on equal ground. One potential improvement of SP (or more accurately, the TCS software) would be the ability to use the statistical parsimony connection limit to connect the less divergent sequences, followed by use of MP to complete the disconnected networks, as was originally proposed by Templeton *et al.* [43].

Finally, the performance of MSN was by far the worst on all simulated data sets. This finding, as pointed out by Cassens *et al.* [12], is likely due to the inability of the minimum spanning network method to infer unsampled historical individuals. The Median Joining Network (RMD) reconstruction method had much better performance than MSN, due to its ability to infer ancestral haplotypes. We strongly discourage the use of MSN for any analyses that rely on the topology of the inferred relationships. Furthermore, when the phylogenetic relationship of any set of sequences is being inferred, it is important that several methods be used and their inferences inspected and compared for discrepancies.

It is clear that there is much room for improvement in the development of methods that infer the historical relationship of intraspecific sequences, particularly when the sequences might have undergone some level of recombination. We look forward to experimenting to increase the accuracy of the existing methods and developing novel methods to more accurately deal with such data.

## Supporting Information

Data S1 Document describing and displaying additional information (Summary statistics of RF and BS from all simulations).
(1.87 MB DOC)
