# Supplementary material for: A Comparison of Phylogenetic Network Methods Using Computer Simulation
Source: PLoS One. 2008 Apr 9;3(4):e1913. doi: 10.1371/journal.pone.0001913 (PMC2275308; doi:10.1371/journal.pone.0001913)

Supplementary Data:

Note on branch score (BS) and robinson-foulds score (RF) normalization:

Since the total number of branches in a bifurcating tree is dependent on the number of tips and this number varied in each set of 1000 simulated histories (because the number of resulting haplotypes can change between replicates), we normalized both the BS and the RF by dividing them by the total number of branches (2N-3 where N is the number of unique OTUs). Thus, the RF and BS values represent the mean difference per branch in each tree.

For measures a-d and 6 (see text), we plotted the distribution for the 1000 replicates using box-and-whisker plots displaying the median, first and second quartiles, and outliers (points further than 3/2 times the inter-quartile range of the first and third quartiles). Additionally, we performed a set of paired Mann-Whitney tests to determine whether the results from each method were significantly different. We used an experiment-wise error rate of 0.05 by correcting the p-value using the Dunn-Sidak multiple test correction. In all of the following figures, the methods and their values are sorted ascending by the mean across 1000 histories. Vertical lines separate two methods when the performance of the two methods was statistically different using a paired Mann-Whitney and a p-value cutoff of .00114.

|  |
| --- |
|  |
|  |
|  |

The figures below should be interpreted with caution, since they are calculated from all trees imbedded in an inferred network, many of which are extremely unlikely given the input data.

**Figure S1**

Mean RF score between each tree in *N* and each tree in *T* (no recombination)


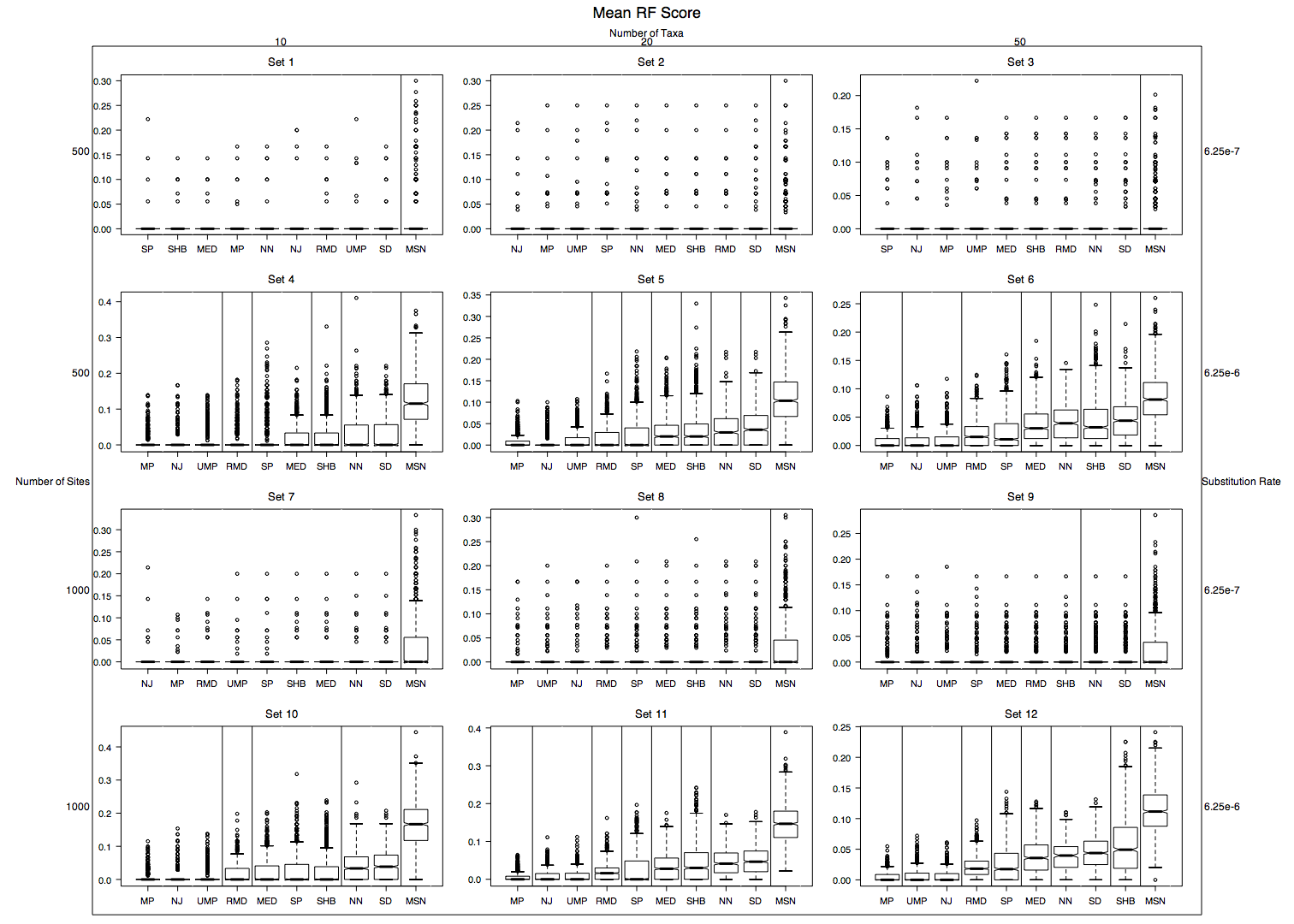


**Figure S2**

Mean BS distance between each tree in N and each tree in T (no recombination)


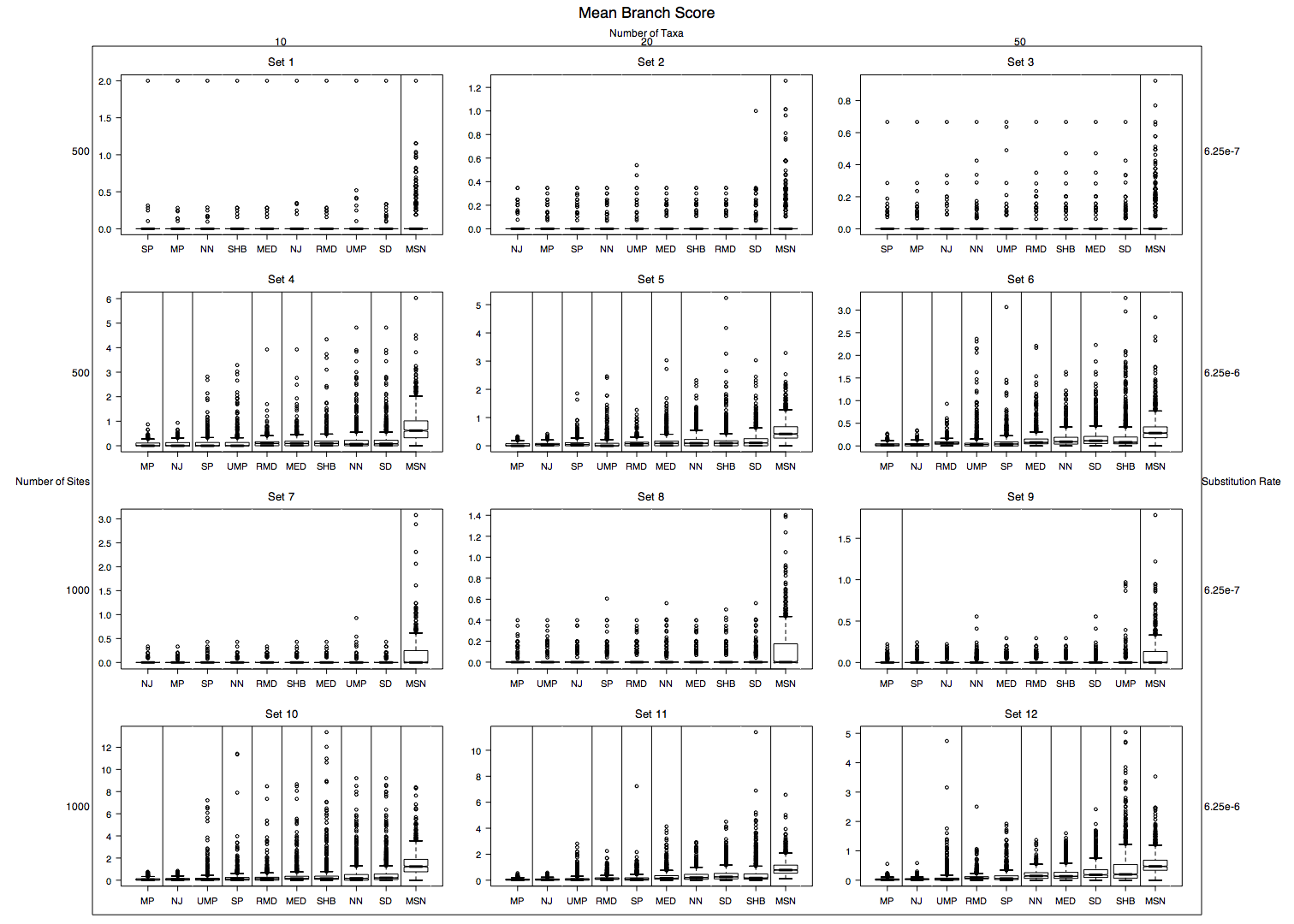


**Figure S3**

Mean RF false positives (no recombination)


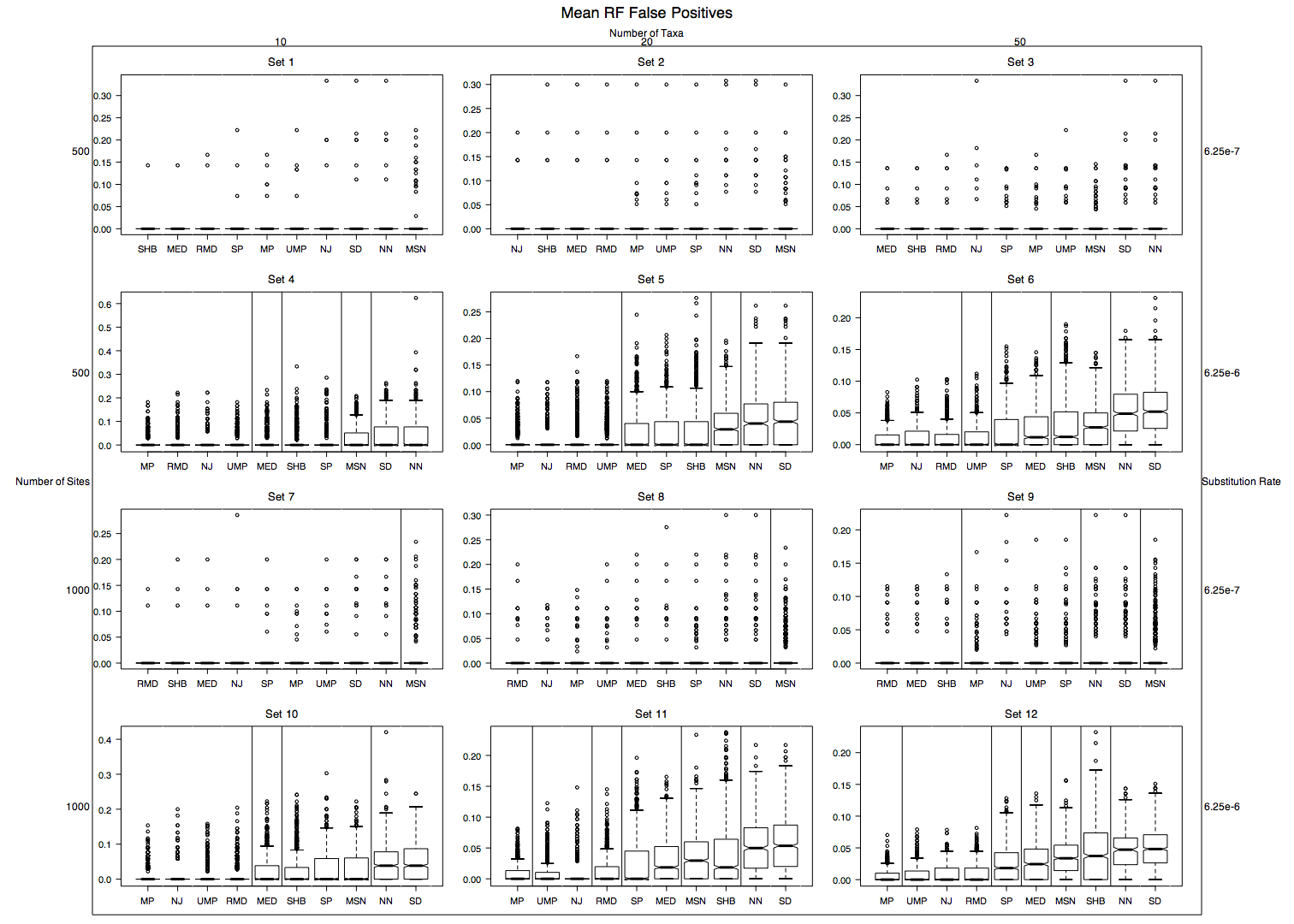


**Figure S4**

Mean RF false negatives (no recombination)


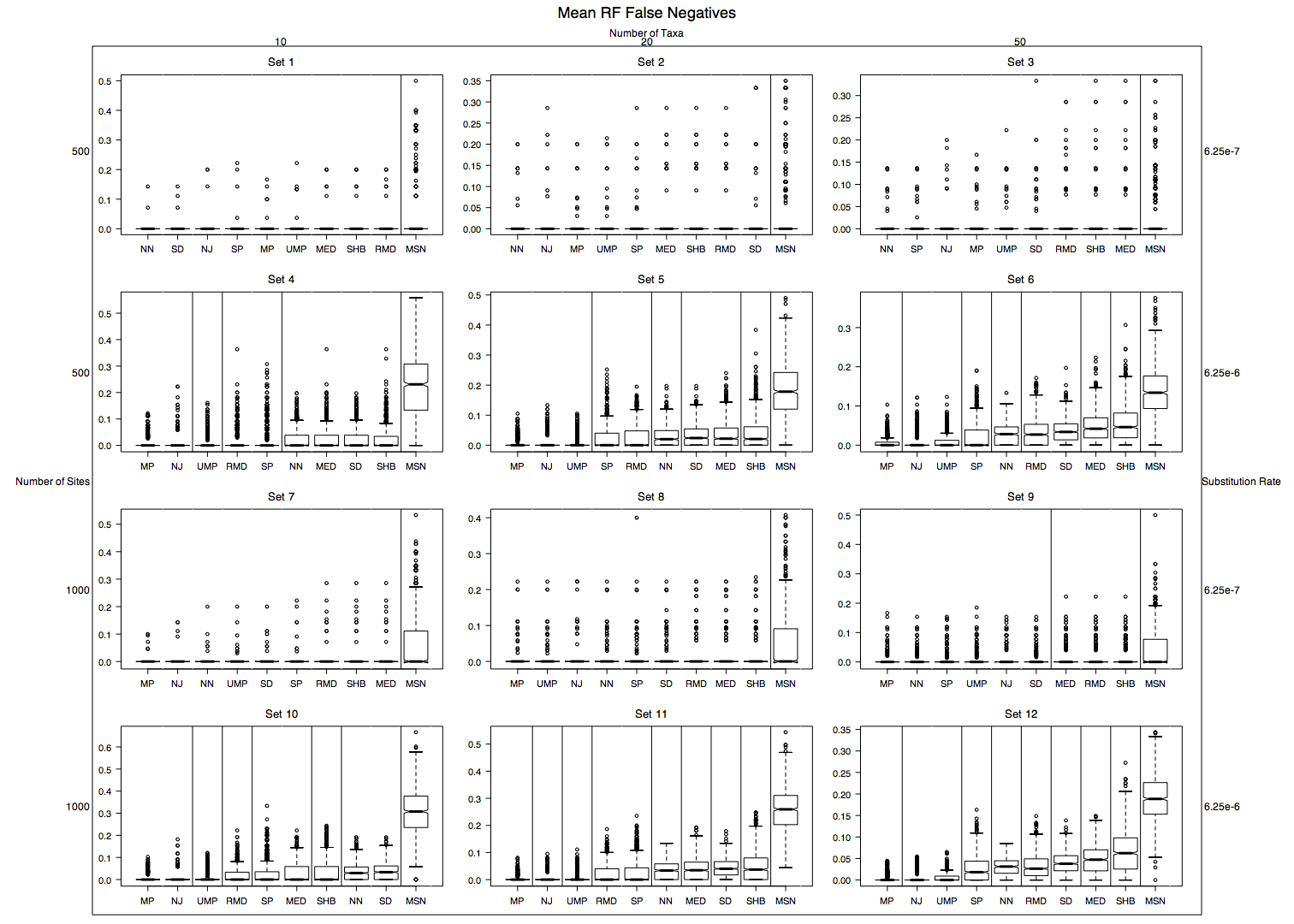


**Figure S5**

Mean BS false positives (no recombination)


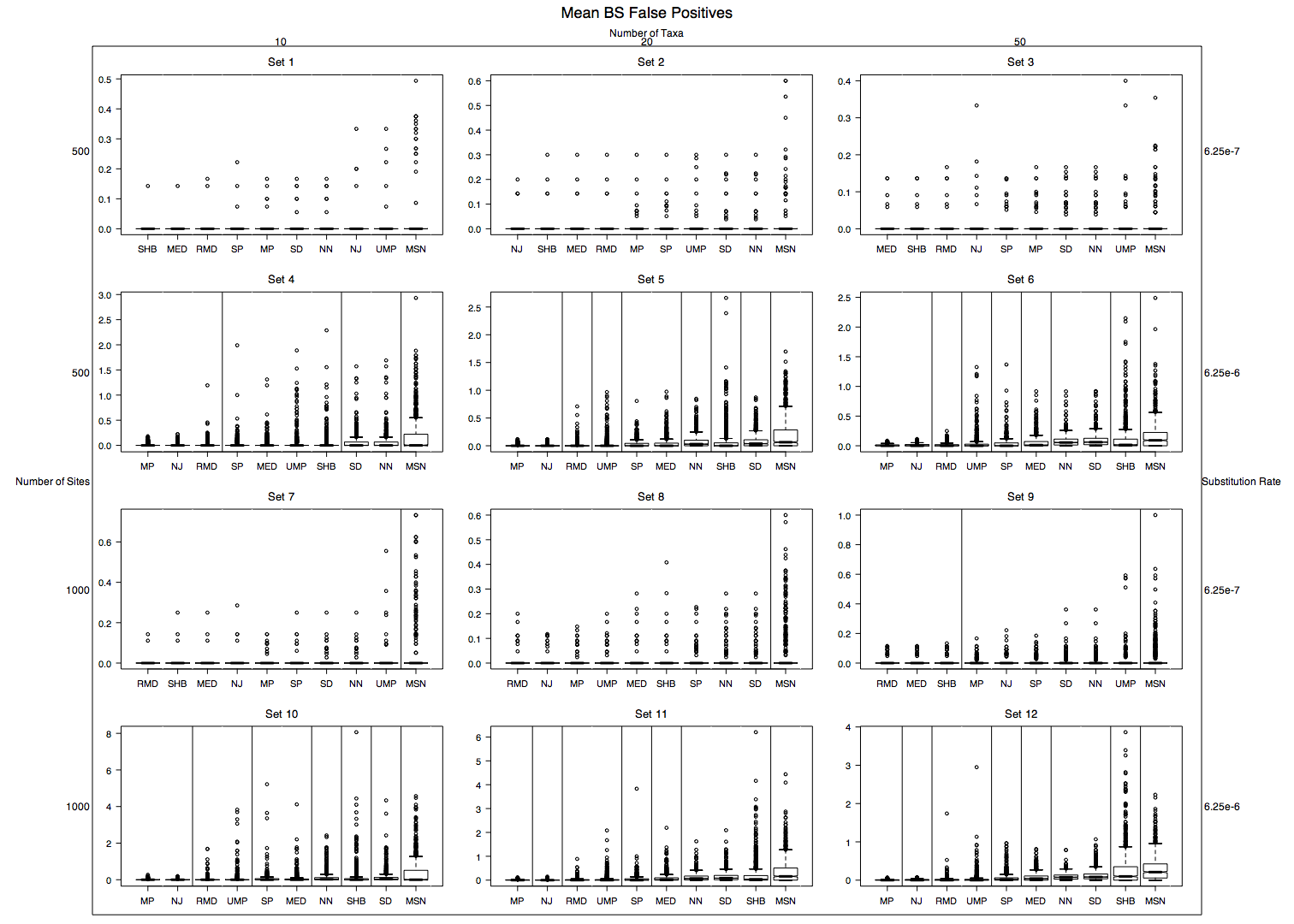


**Figure S6**

Mean BS false negatives (no recombination)


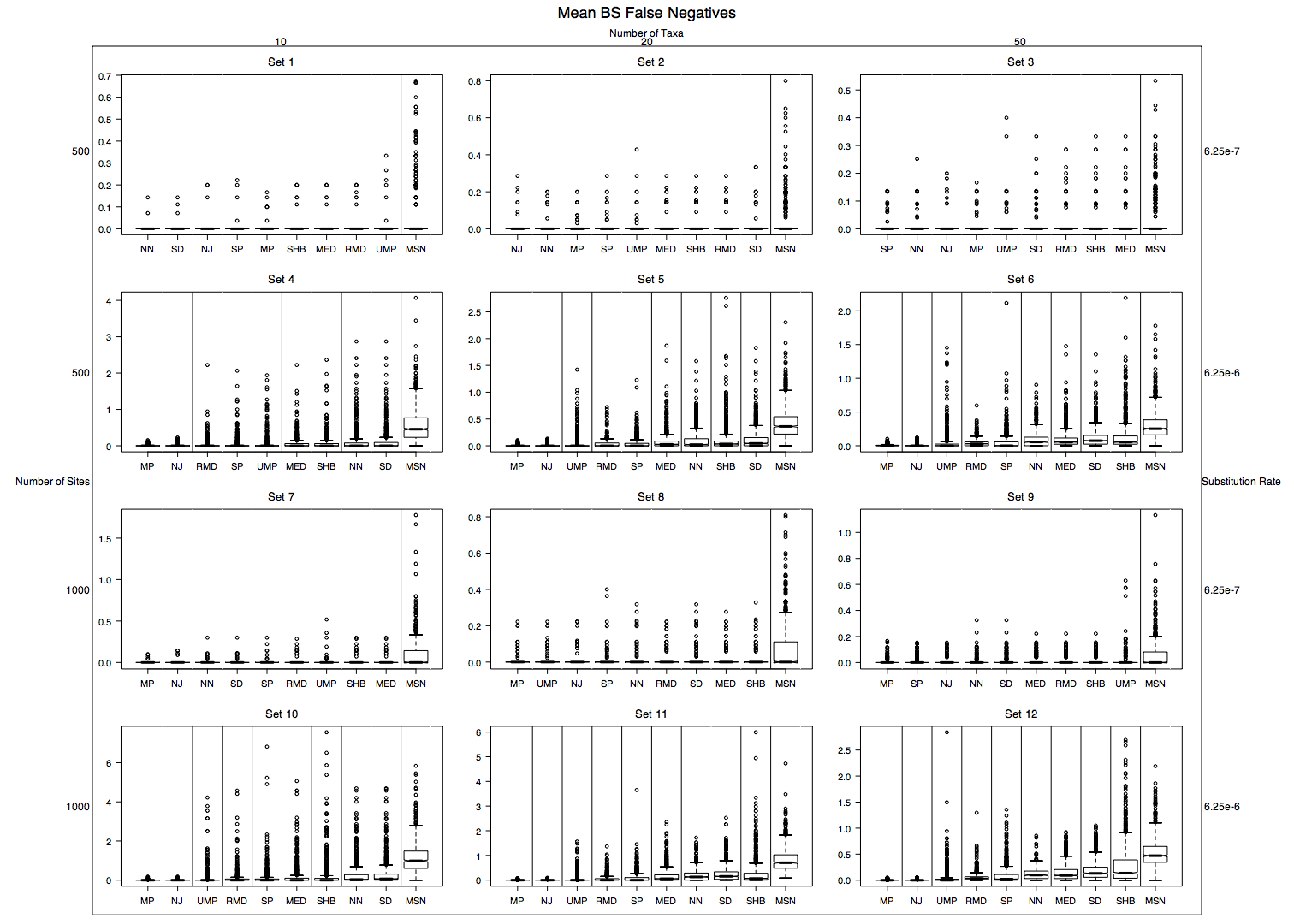


**Figure S7**

Mean branch length difference between matching branches (no recombination)


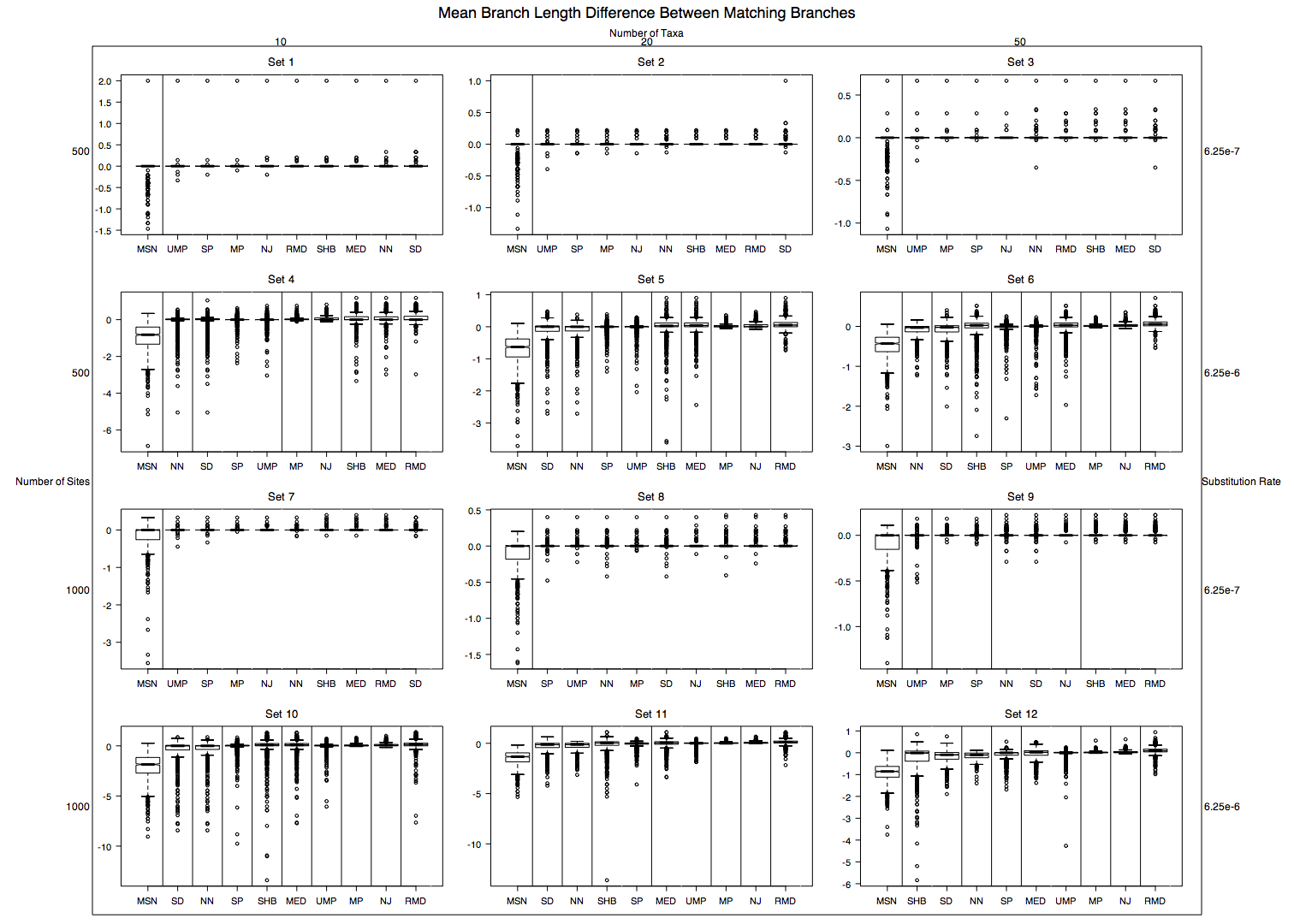


Supplementary Information:

The figures below should be interpreted with caution, since they are calculated from all trees imbedded in an inferred network, many of which are extremely unlikely given the input data.

Note on branch score (BS) and robinson-foulds score (RF) normalization:

Since the total number of branches in a bifurcating tree is dependent on the number of tips and this number varied in each set of 1000 simulated histories (because the number of resulting haplotypes can change between replicates), we normalized both the BS and the RF by dividing them by the total number of branches (2N-3 where N is the number of unique OTUs). Thus, the RF and BS values represent the mean difference per branch in each tree.

For measures a-d (see text), we plotted the distribution for the 1000 replicates using box-and-whisker plots displaying the median, first and second quartiles, and outliers (points further than 3/2 times the inter-quartile range of the first and third quartiles). Additionally, we performed a set of paired Mann-Whitney tests to determine whether the results from each method were significantly different. We used an experiment-wise error rate of 0.05 by correcting the p-value using the Dunn-Sidak multiple test correction. In all of the following figures, the methods and their values are sorted ascending by the mean across 1000 histories. Vertical lines separate two methods when the performance of the two methods was statistically different using a paired Mann-Whitney and a p-value cutoff of .00114.

|  |
| --- |
|  |
|  |

**Figure S8**

Mean RF score between each tree in *N* and each tree in *T* (recombination)


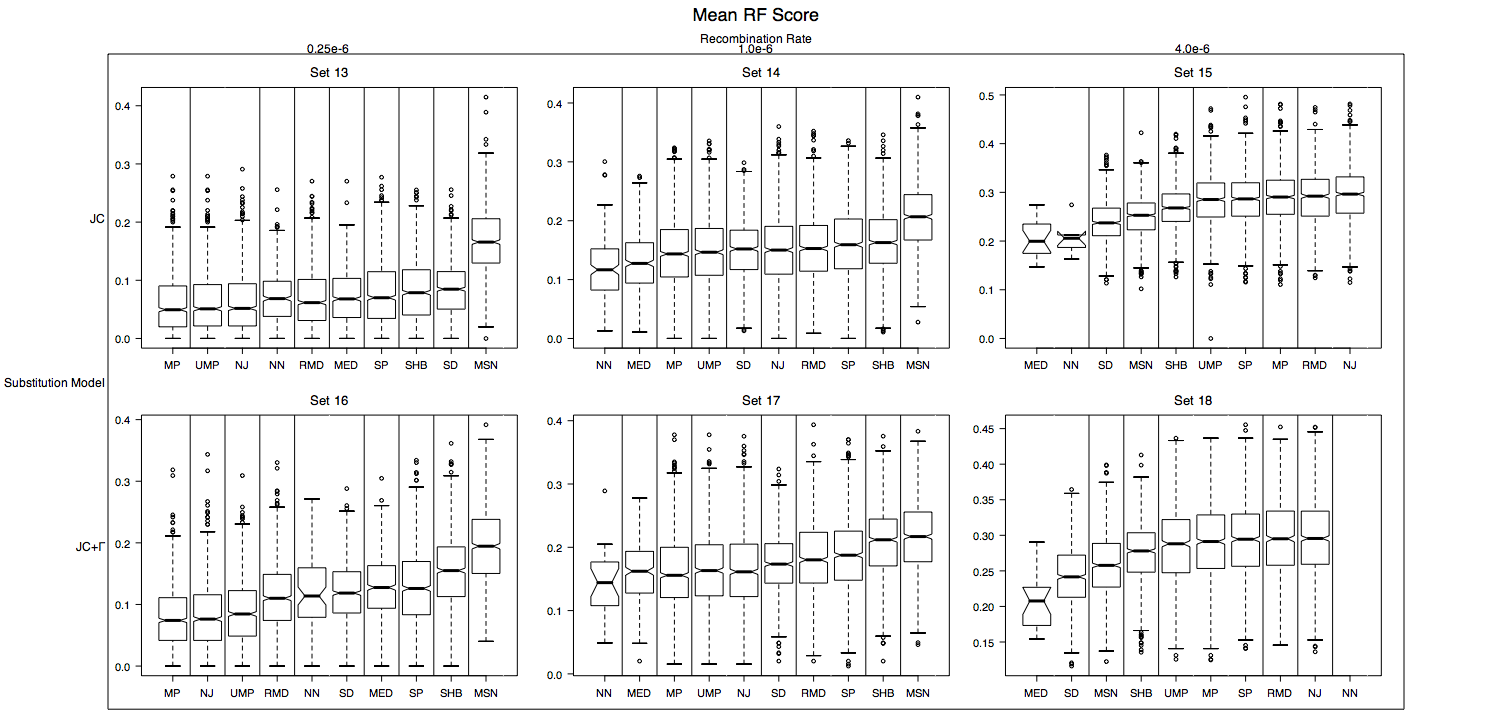


**Figure S9**

Mean BS distance between each tree in N and each tree in T (recombination)


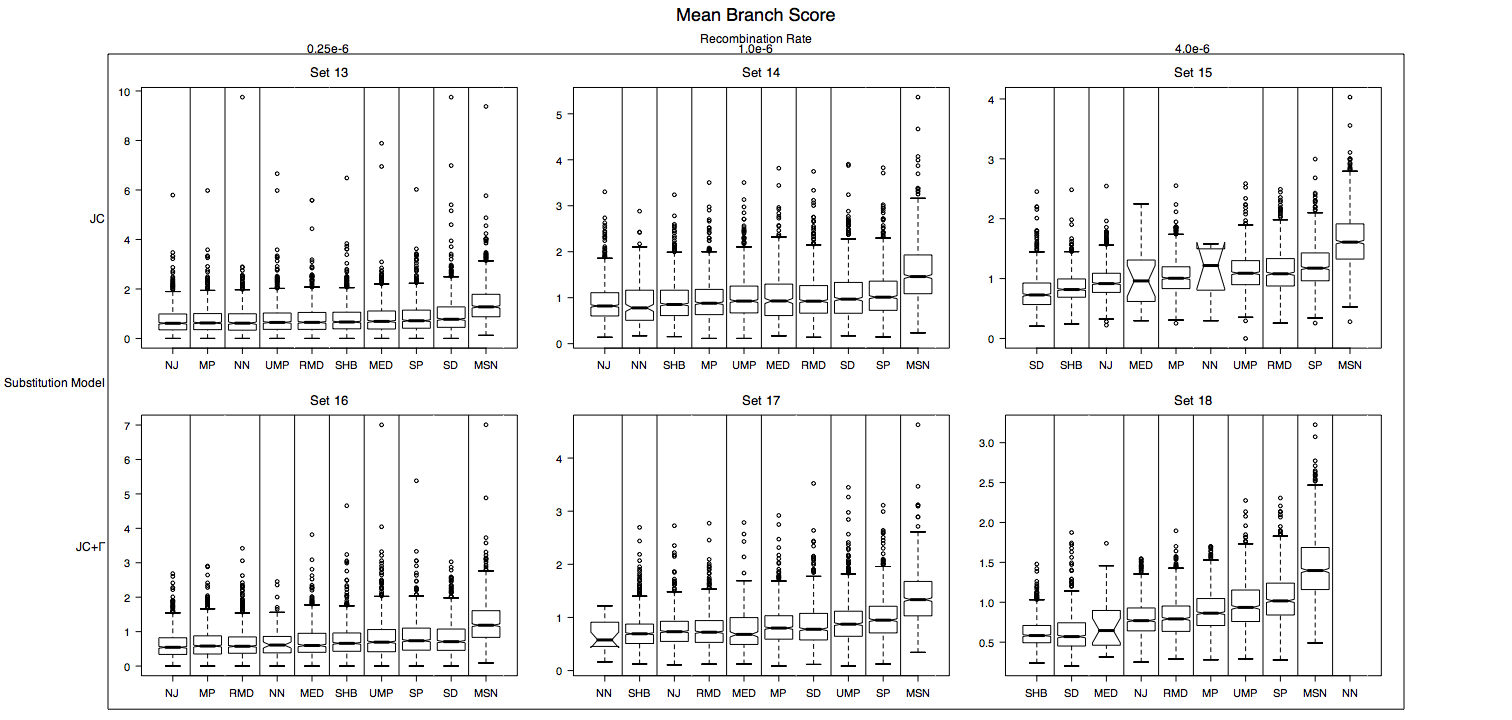


**Figure S10**

Mean RF false positives (recombination)


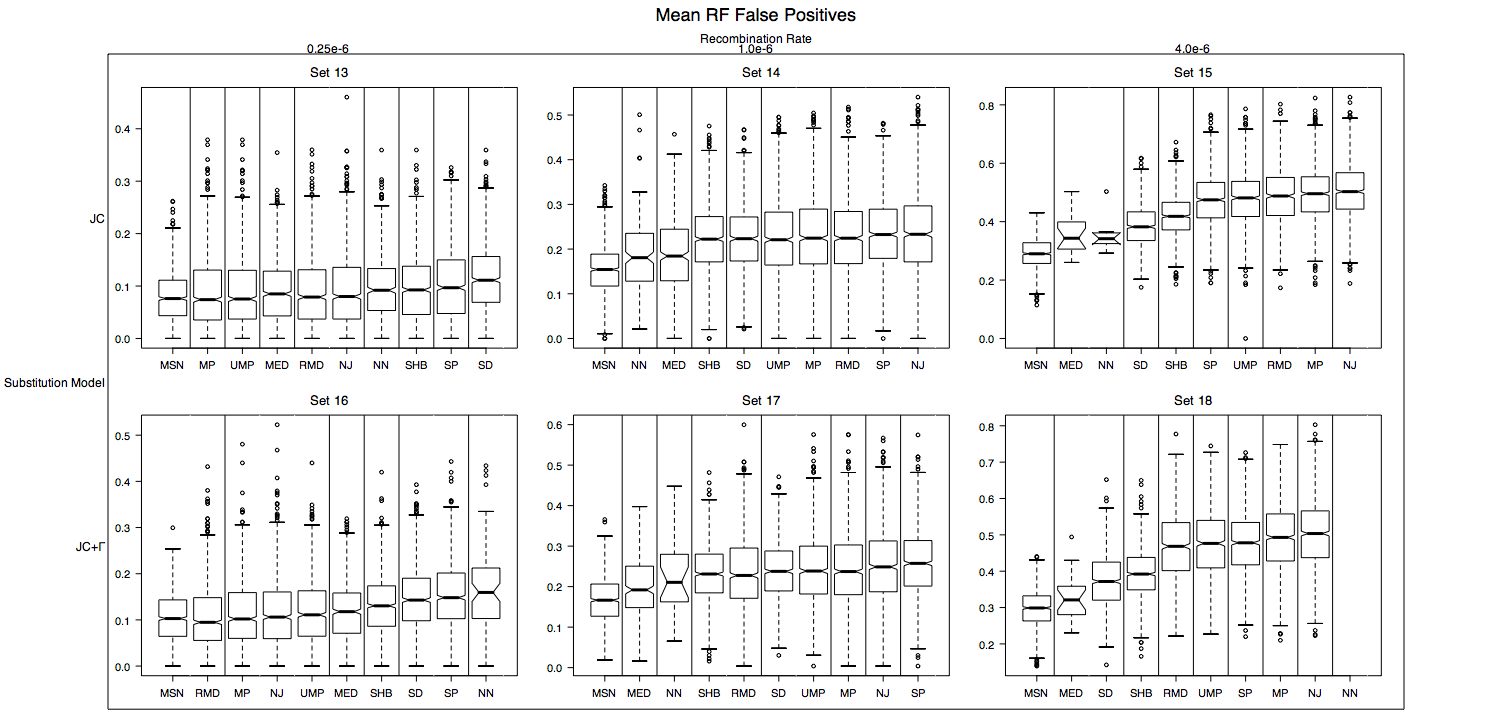


**Figure S11**

Mean RF false negatives (recombination)


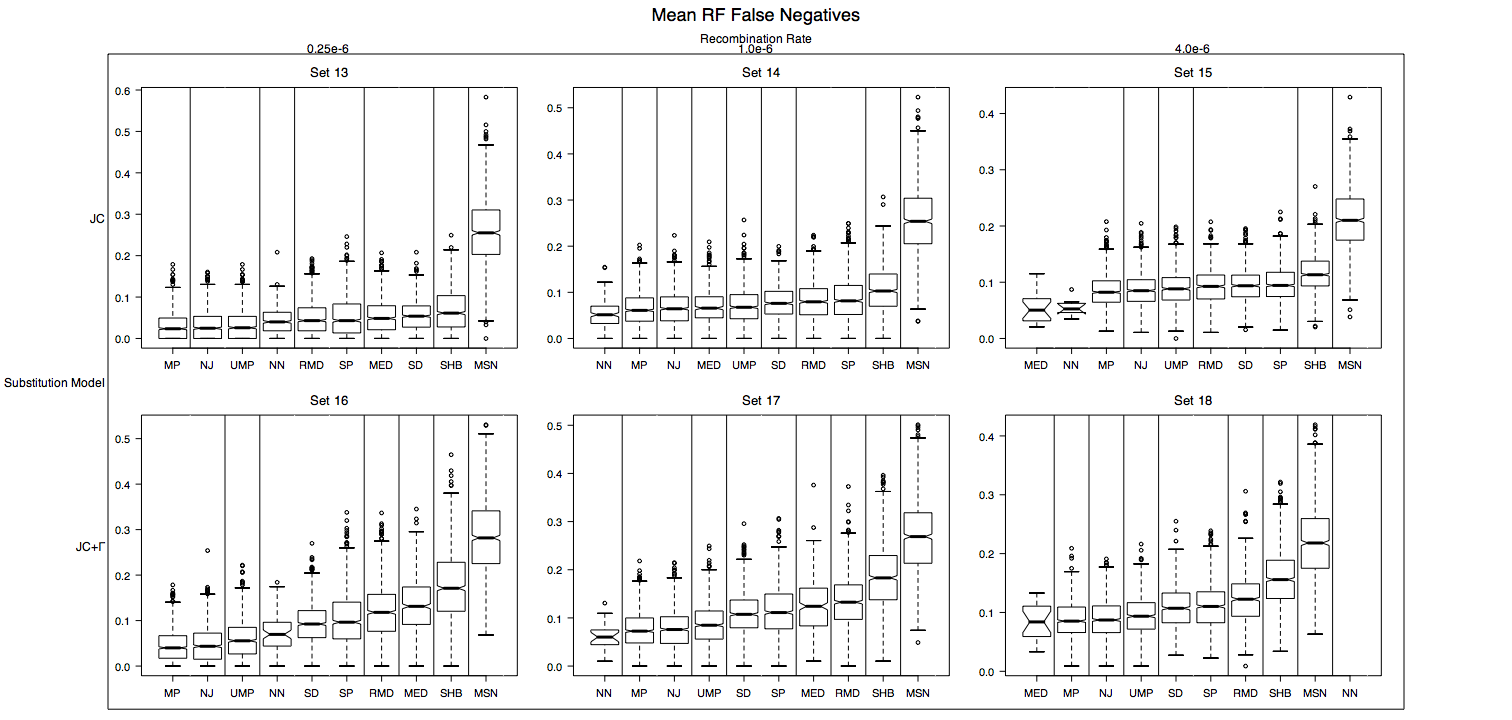


**Figure S12**

Mean BS false positives (recombination)


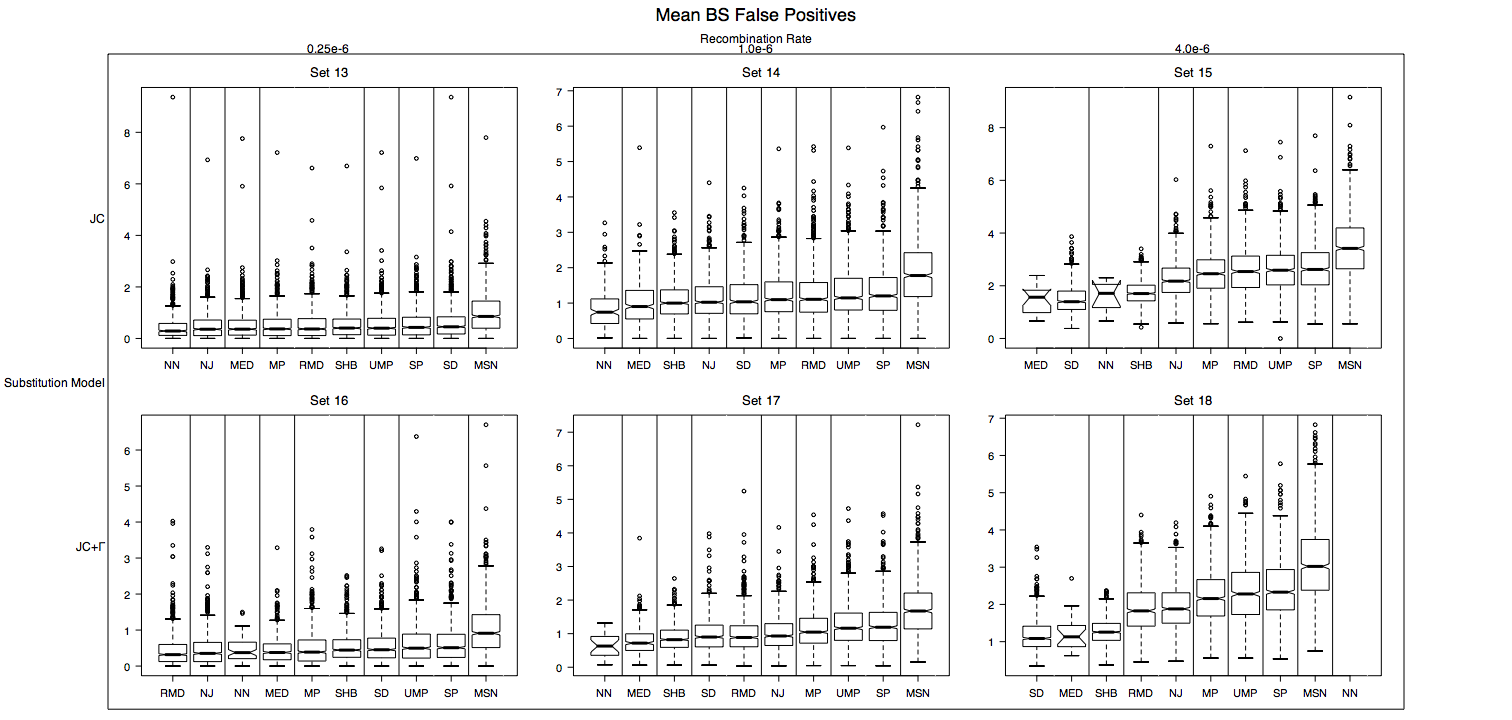


**Figure S13**

Mean BS false negatives (recombination)


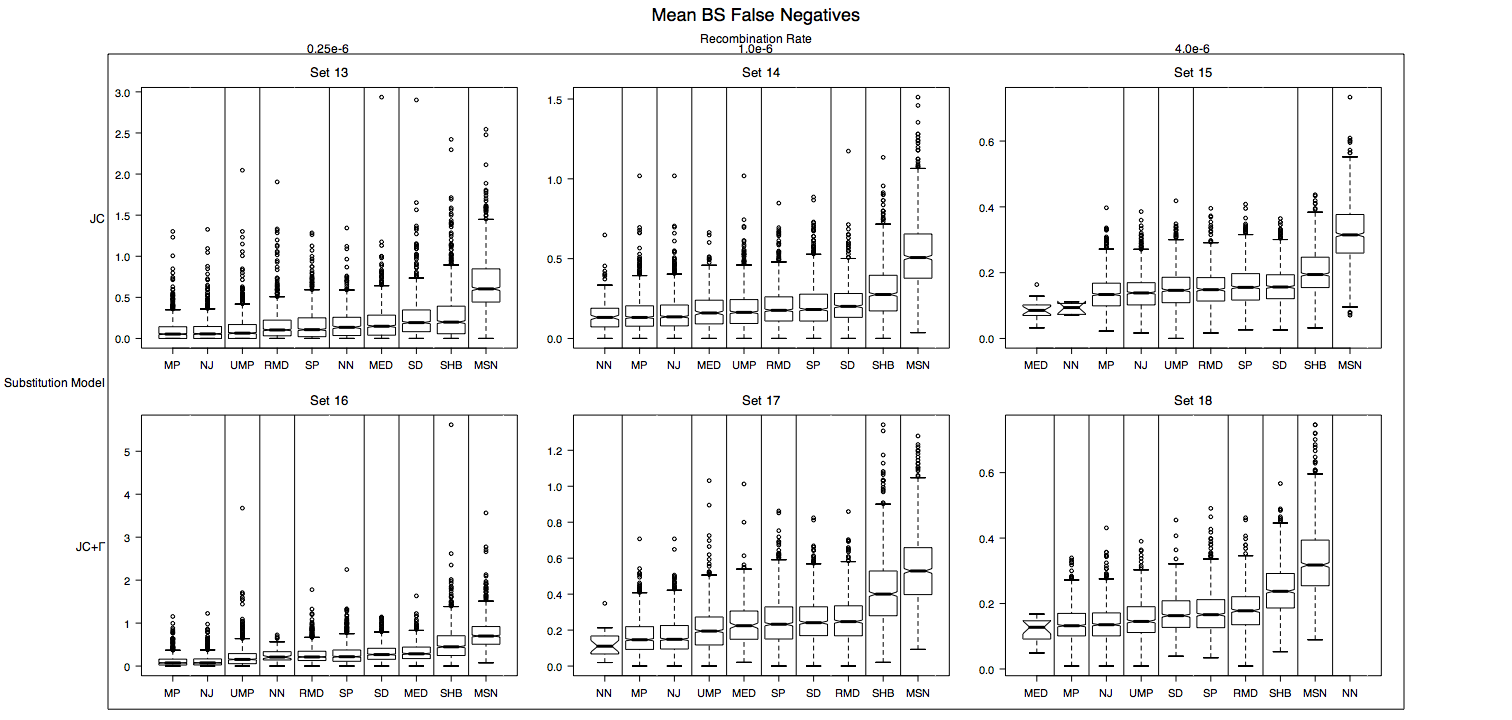

Supplement: Data S1 — Document describing and displaying additional information (Summary statistics of RF and BS from all simulations). (1.87 MB DOC) [file pone.0001913.s001.doc]
